# Avoidable Compartment Syndrome! High Index of Suspicion for a Newly Presenting Haemophiliac: A Case Series

**DOI:** 10.1155/2016/3263261

**Published:** 2016-05-31

**Authors:** A. Niblock, K. Donnelly, F. Sayers, P. Winter, G. Benson

**Affiliations:** ^1^Northern Ireland Haemophilia Comprehensive Care Centre, Belfast City Hospital, Lisburn Road, Belfast BT10 7AB, UK; ^2^Department of Orthopaedics, Royal Victoria Hospital, Grosvenor Road, Belfast BT12 6BA, UK; ^3^Department of Physiotherapy, Belfast City Hospital, Lisburn Road, Belfast BT10 7AB, UK; ^4^Genetics Department, Belfast City Hospital, Lisburn Road, Belfast BT9 7AB, UK

## Abstract

Bleeding disorders can present at any age and vary in their severity. Haemophilia, which is characterised by its x-linked recessive inheritance, can present with a spontaneous mutation and therefore no family history will be evident. Three cases of trauma induced thigh haematomas as an initial presenting feature for people with haemophilia are discussed. The cases highlight the importance of a coagulation screen if the patients bleeding phenotype does not match the injury sustained. An isolated prolonged APTT with no offending anticoagulant cause should always be investigated to look for underlying haemophilia. Interestingly the cases demonstrate the limitations of a coagulation screen. Factor VIII being an acute phase reactant can result in the fact that the initial coagulation screen may be temporarily normal. Therefore, if there is a high index of suspicion for a bleeding disorder, consider repeating the coagulation screen and seeking haematology opinion. Early diagnosis and appropriate specific factor replacement for an injured haemophiliac prevent haematomas expanding thus avoiding potential complications like compartment syndrome or unnecessary surgical input.

## 1. Introduction

When vasculature becomes compromised, the body triggers an integrated cascade involving the vessel wall, platelets, and coagulation factors. The intention is to achieve haemostasis, eventually aiming to restore the vessel calibre to normal. Abnormal bleeding phenotypes can be caused by defects within the vessel wall, coagulation factor deficiencies, or platelets abnormalities like thrombocytopenia or platelet function disorders.

Severe bleeding disorders are easier to diagnose, as physicians can see the extent of bleeding and generally they present at a younger age. Milder bleeding disorders are a neglected diagnosis because they can be a diagnostic challenge. Some are discovered through a coincidental coagulation screen and a retrospective mild bleeding history. Others can present to adult services later in life usually after a provoked bleed from trauma or surgical procedures.

When a patient presents with a bleed, the examining physician must ensure the extent of the bleeding is in keeping with the mechanism of injury sustained. A thorough bleeding history including family history at presentation can also highlight an underlying problem. The following cases stress the diagnostic difficulty and late presentation of mild haemophilia. Early diagnosis can prevent unnecessary complications and interventions and shorten hospital admissions. At-risk patients can be identified, providing them with appropriate counselling and future preventative management strategies.

## 2. Case Reports

### 2.1. Case  1

In March 2013, Mr. A, a 25-year-old male, was playing football when another player's knee collided with his thigh. He remembered immediate pain and swelling over his upper quadriceps. However, he was able to continue playing and later that evening the pain increased in severity. Mr. A was unable to sleep and the next morning he presented to the university sports injury clinic. At this stage, examination noted visible swelling but no neurological or vascular compromise. Initial treatment consisted of rest, ice, and analgesia and the patient was discharged with crutches.

Unfortunately, 5 days later, Mr. A returned with increased swelling, bruising, and paraesthesia. He was referred to the emergency department for further assessment.  Figure 3 shows the extent of the thigh swelling. His routine blood tests revealed haemoglobin of 80 g/L (normal 135 g/L–175 g/L). CT scan of thigh estimated the haematoma volume at >2 L and given his early neuropathy he was referred for urgent fasciotomy.

After fasciotomy (Figure 4), Mr. A required two units of packed red cells. A coagulation screen preoperatively showed normal PT and APTT and increased fibrinogen. Six days after fasciotomy closure, Mr. A re-presented with swelling and a discharging wound. The wound was evacuated and further packed red cells were transfused. Another coagulation screen was performed prior to skin grafting, which revealed a prolonged APTT of 34 seconds (normal range 21–32 seconds).

Further investigations by haematology revealed a low Factor IX of 27.2% in keeping with a diagnosis of mild haemophilia B. Prophylactic enoxaparin was discontinued and regular tranexamic acid was started. Mr. A made a full recovery within 2 months and is now under the care of a comprehensive care haemophilia centre. He had extended family screening confirming his mother and maternal grandmother as carriers of haemophilia as well as a maternal aunt whose son was confirmed to have haemophilia.

### 2.2. Case  2

In July 2004, Mr. L, a 29-year-old male, suffered from a left thigh injury playing football and presented the next day to the emergency department. It was noted that he had increased swelling and paraesthesia and was unable to weight-bear.

On further questioning, this gentleman had a history of recurrent haematomas presenting in 1994 and again in 1998. No surgical intervention was required on these occasions. He was previously surgically challenged undergoing a tonsillectomy as a child with no haemorrhagic complications. Mr. L remembers suffering multiple epistaxis as a teenager but never required hospital admissions. He gave no family history of any bleeding disorders on direct questioning.

On this occasion, Mr. L was treated conservatively with bed rest, analgesia, neurovascular observations, and serial ultrasounds of the haematoma. Mr. L received prophylactic enoxaparin during admission for his VTE risk. The haematoma was slow to resolve resulting in a prolonged hospital admission.

Coagulation screen persistently revealed elevated APTT for 44 seconds (normal range 21–32 seconds) and therefore haematology was contacted. After the 50 : 50 plasma mix showed correction of the APTT, intrinsic factors were quantified. Factor VIII was low at 24% and VW antigen and activity were appropriate for his blood group. The diagnosis was in keeping with mild haemophilia A and he is under the long-term care of a comprehensive care haemophilia centre. Mr. L was from a family of 11 siblings and extended family genetic screening revealed that 4 of his brothers also had undiagnosed mild haemophilia A and two of his sisters were carriers. The patient returned to sport after 1 month of outpatient physiotherapy and is now treated with on-demand factor replacement.

### 2.3. Case  3

In February 2010, Mr. M, a 27-year-old male, had a similar injury mechanism as discussed in Case  1, again playing football. He presented to the emergency department with pain and swelling and was at this stage neurovascularly intact. However, over the next 24 hrs his haematoma expanded and he underwent a fasciotomy due to neurovascular compromise. He was referred to haematology with isolated prolonged APTT of 40.2 seconds (normal range 21–32 seconds) and was diagnosed with mild haemophilia A.

One week later, Mr. M returned with increased pain and swelling. Mr. M received recombinant Factor VIII preoperatively and underwent a femoral arteriogram plus embolisation for two pseudoaneurysms (Figure 5). His haematoma was evacuated and a drain was inserted. Mr. M underwent extensive family screening revealing another brother and cousin with haemophilia. He currently remains active playing multiple sports. His current management strategy after minor trauma includes self-medicating with DDAVP. Mr M regularly attends the haemophilia outpatient department were his concerns are addressed by the multidisciplinary team (Table 1).

## 3. Discussion

Acute compartment syndrome is an orthopaedic emergency that is most commonly seen in the lower leg, although it may occur throughout both the upper and the lower limbs. Often there is an underlying fracture, although compartment syndrome can be seen in the setting of crush injuries, reperfusion, and muscle injury. The underlying process is a progressively increasing pressure in a closed osteofascial compartment, which if left untreated can result in inadequate muscle perfusion causing ischaemia and necrosis [1]. Nerves in the compartment can also be affected by the rising pressure and hypoperfusion, although this is often a late finding. Diagnosis of compartment syndrome is often clinical, with pain out of proportion with the injury, exacerbated by passive movements, which responds poorly to opiate analgesia [2]. Once a diagnosis of acute compartment syndrome has been made, the patient should undergo immediate decompression to preserve as much tissue as possible.

Compartment syndrome of the thigh is uncommon, especially in the absence of a concomitant femoral fracture. A recent paper by Ojike et al. [3] reviewed 89 cases reported in the literature. 85% of patients were male with an average age of 38 years. Almost all cases (90%) were associated with blunt trauma, with 44 patients having an associated femoral fracture. Nineteen patients in this review had coagulation deficiencies such as DIC, von Willebrand disease, and therapeutic anticoagulation.

Haemophilia A and haemophilia B are bleeding disorders, inherited in an x-linked recessive fashion, caused by deficiencies in Factors VIII and IX, respectively. The incidence of haemophilia A is 1 : 5000 and that of haemophilia B is 1 : 30000 live male births. The genes associated with these conditions are located on the X chromosome. In males, one altered copy of the gene in each cell is sufficient to cause the condition. In x-linked recessive inheritance, a female with one altered copy of the gene in each cell is called a carrier. Carrier females have about half the usual amount of coagulation factor, which is generally enough for normal blood clotting. However, about 10 percent of carrier females have less than half the normal amount of one of these coagulation factors; these individuals are at risk for abnormal bleeding, particularly after an injury, surgery, or tooth extraction [4].

The symptoms of haemophilia vary depending on how severe the condition is, but the main sign is prolonged bleeding. The severity of the condition is determined by the level of clotting factors in the blood: mild haemophilia, 5%–50% of the normal amount of clotting factor, may not have any symptoms for many years. The condition usually only becomes apparent after a significant wound, surgery, or a dental procedure such as having a tooth removed. Moderate haemophilia, 1%–5% of the normal amount of factor, may bruise easily and have symptoms of internal bleeding around their joints, particularly if they have a knock or a fall that affects their joints. Severe haemophilia is characterised by a factor level less than 1%. As a result bleeding in joints is more severe and frequent.

A coagulation screen consists of the prothrombin time (PT), activated partial thromboplastin time (APTT), and fibrinogen assay. It was designed to screen a bleeding patient for a coagulation factor deficiency. It was not intended as a test for haemostasis and a normal coagulation screen will not ensure haemostasis. The most important factor to determine patients bleeding risk is a detailed bleeding history. A history of recurrent prolonged bleeding from various sources suggests abnormal haemostasis. Recurrent localised bleeding would indicate a local anatomical problem. If the bleeding history is suggestive or the bleeding is exaggerated for injury sustained, it is reasonable to perform a coagulation screen. The coagulation screen is an in vitro test designed to look for possible factor deficiencies. It is important to remember Factor VIII is an acute phase reactant and may increase after trauma. A temporary increase in Factor VIII level may normalise the APTT masking a mild haemophilia A or even a mild haemophilia B demonstrated in Case  1. A coagulation screen will also be normal with platelet function disorders such as certain types of von Willebrand disease and FXIII deficiency. FXIII deficiency has a severe bleeding phenotype usually diagnosed at birth after prolonged bleeding from the clamped umbilical cord [5].

**Figure 1:**
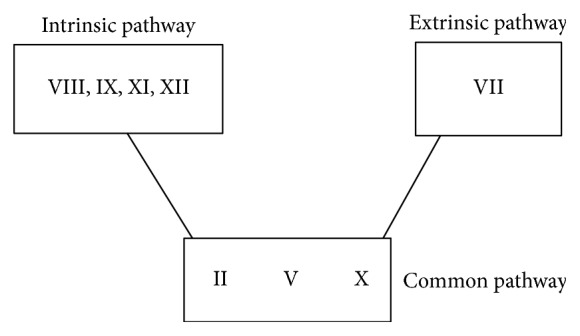


As the coagulation screen has limitations, the bleeding history is the most appropriate screen and a suggestive history should be investigated by the haematology team (see Figure 1).

The PT represents the extrinsic pathway consisting of Factors VII, II, V, and X. The APTT represents the intrinsic pathways VIII, IX, XI, XII, II, V, and X. Common to both these pathways are Factors II, V, and X. A deficiency of the common factors would usually present with prolonged APTT and PT.

**Figure 2:**
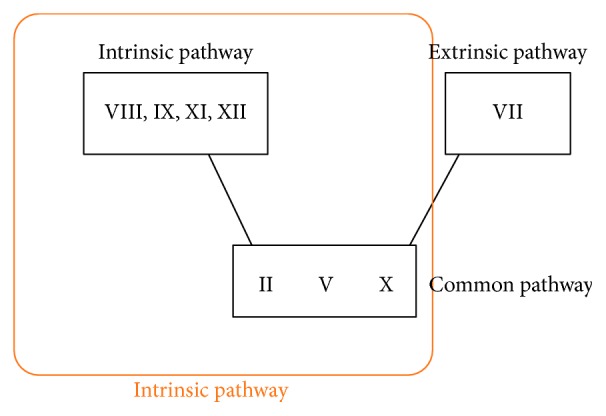


**Figure 3:**
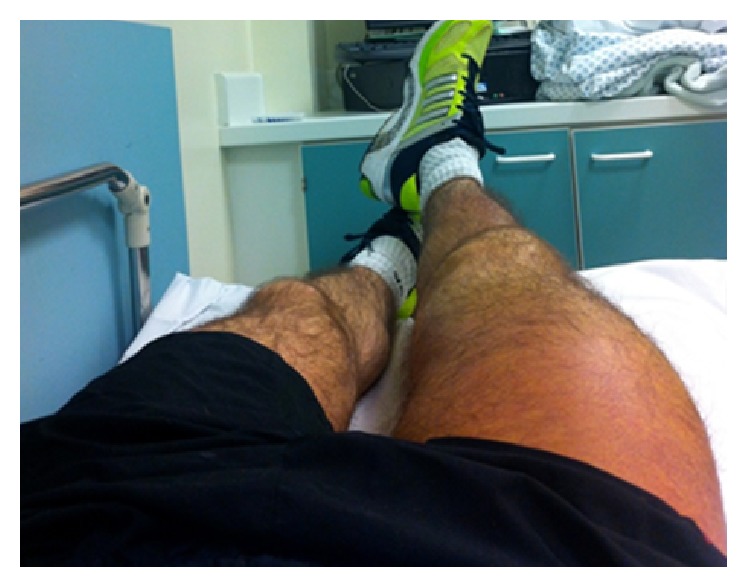
Extent of thigh swelling 5 days after initial trauma, Case  1.

**Figure 4:**
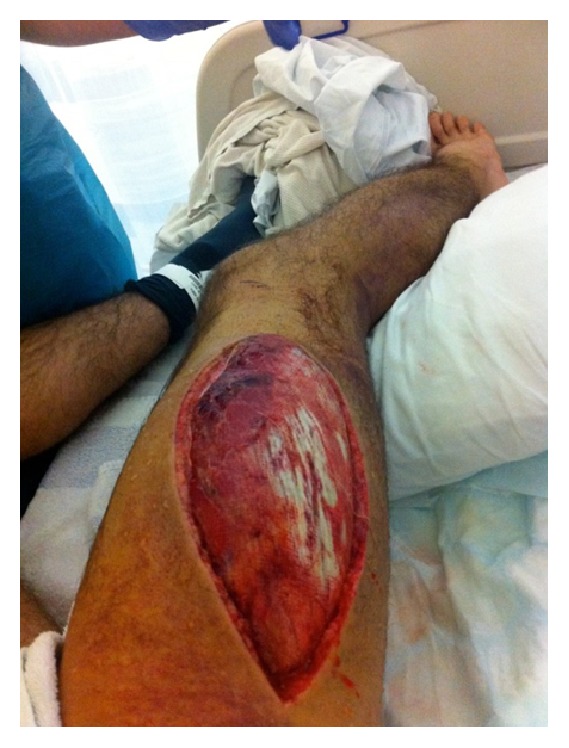
Immediately after fasciotomy, Case  1.

**Figure 5:**
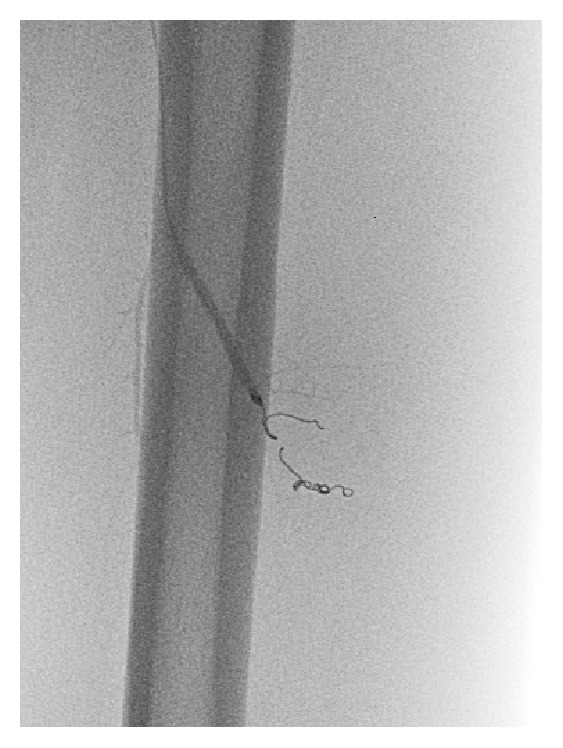
An area of definite vascular abnormality is demonstrated in the deep muscular branches of the profunda and proximal thigh. This correlated with the large expanding haematoma. A segment of these distal vessels was embolised using several coils.

In each of the three case studies, the patients eventually had an isolated prolonged APTT. The coagulation screens had a normal PT, indicating Factor VII, and the common pathway Factors II, V, and X should all be normal. The next step to investigate the prolonged APTT is to perform a 50 : 50 mix where 50% of patients' plasma is added to normal plasma. If the APTT corrects, this indicates a factor deficiency. Therefore, the only accountable factors should be VIII, IX, XI, and XII. Factor XII deficiency is not associated with a bleeding phenotype. Individual factor assays will determine which factor is responsible for the prolonged APTT (see Figure 2).

Interestingly, Case  1 initially had a normal APTT on the coagulation screen. It was only after a repeat coagulation screen revealed a prolonged APTT did he get diagnosed with mild haemophilia B. This Factor IX deficiency was masked as FVIII is an acute phase reactant and its elevated levels compensated for the in vitro APTT [6].

Case three demonstrates the importance of making the diagnosis. When Mr. M was readmitted and treated with appropriate factor replacement, he had no operative or postoperative complications. Early diagnosis and prompt treatment of a patient with haemophilia may prevent a haematoma progressing into compartment syndrome. This may prevent unnecessary surgery and the associated complications.

Depending on the severity of the haemophilia, there are various treatment options. Severe haemophiliacs with factor levels <1% are maintained on prophylactic factor replacement. Mild haemophiliacs > 5% usually do not require regular treatment and depending on the bleed/injury they can be treated with on-demand factor replacement, DDAVP, or tranexamic acid. A comprehensive care haemophilia centre regularly reviews patients with haemophilia. These centres specialise in providing a network of support for the patients as well as medical professionals with the aim of preventing and dealing with potential bleeding risks. The three cases described above made a full physical recovery after an appropriate course of physiotherapy organised through the haemophilia centre. Having access to a physiotherapist who specialises in haemophilia is essential for optimal and safe management of joint and muscle bleeds. A physiotherapist will be able to provide information on how to improve joint mobility and muscle health and give bleed preventative advice and treatment after an acute bleeding episode. After a joint or muscle bleed, the affected area should be rested and physiotherapy treatment should begin after bleeding has been fully controlled. Regular physiotherapy assessment can play a part in reducing the effects of bleeding into joints, which may lead to permanent damage and the need for surgery [7].

**Figure 6:**
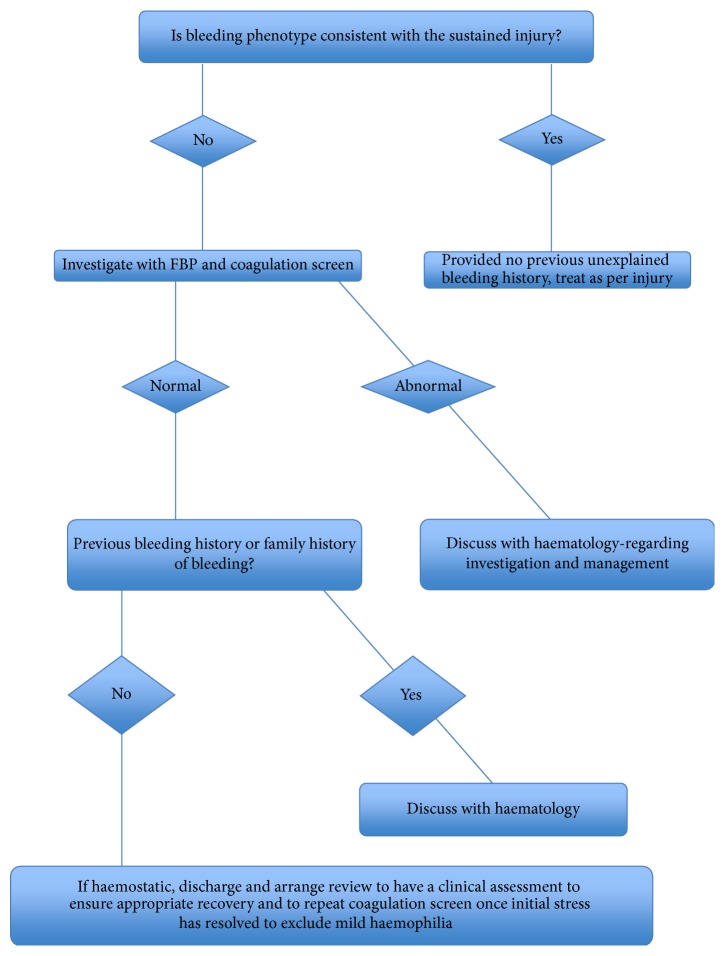
Proposed strategy for emergency departments to maximise chances of diagnosing mild haemophilia.

Figure 6 demonstrates a proposed strategy for Emergency Departments to maximise chances of diagnosing mild haemophilia.

To summarise, this case series highlights several important points. When anyone presents with a bleed or haematoma unusual for the injury sustained, a bleeding history including family history is the most important initial step. If suspicion is high for a bleeding disorder, a coagulation screen is warranted, bearing in mind it has limitations. It can help guide further investigations but will not exclude all bleeding disorders. An isolated prolonged APTT should alert the clinician to a possible type of underlying haemophilia and should be promptly investigated. Diagnosis of new haemophilia triggers family genetic screening identifying other at-risk family members. Early diagnosis with appropriate management can prevent further damage, reducing the need for surgical intervention and potentially reducing hospital admissions.

## Figures and Tables

**Table 1:** Summary of cases.

	Mechanism	Sport	Age	Diagnosis	Level	Prior history of haematoma	Family history
Case 1	Provoked-direct player contact	Soccer	26	Mild B	0.28 IU/mL	No	Not on initial history taking, but retrospective familial genetic studies positive

Case 2	Provoked-direct player contact	Soccer	29	Mild A	0.24 IU/mL	Yes	Not on initial history taking, but retrospective familial genetic studies positive

Case 3	Provoked-direct player contact	Soccer	27	Mild A	0.28 IU/mL	Yes	Not on initial history taking, but retrospective familial genetic studies positive
